# Local adaptation stops where ecological gradients steepen or are interrupted

**DOI:** 10.1111/eva.12789

**Published:** 2019-04-26

**Authors:** Jon R. Bridle, Masakado Kawata, Roger K. Butlin

**Affiliations:** ^1^ School of Biological Sciences University of Bristol Bristol UK; ^2^ Graduate School of Life Sciences Tohoku University Sendai Japan; ^3^ Department of Animal and Plant Sciences University of Sheffield Sheffield UK; ^4^ Department of Marine Science University of Gothenburg Gothenburg Sweden

**Keywords:** ecological margins, local adaptation, niche expansion, patchiness, population genetics

## Abstract

Population genetic models of evolution along linear environmental gradients cannot explain why adaptation stops at ecological margins. This is because, unless models impose reductions in carrying capacity at species’ edges, the dominant effect of gene flow is to increase genetic variance and adaptive potential rather than swamping local adaptation. This allows the population to match even very steep changes in trait optima. We extend our previous simulations to explore two nonlinear models of ecological gradients: (a) a sigmoid (steepening) gradient and (b) a linear gradient with a flat centre of variable width. We compare the parameter conditions that allow local adaptation and range expansion from the centre, with those that permit the persistence of a perfectly adapted population distributed across the entire range. Along nonlinear gradients, colonization is easier, and extinction rarer, than along a linear gradient. This is because the shallow environmental gradient near the range centre does not cause gene flow to increase genetic variation, and so does not result in reduced population density. However, as gradient steepness increases, gene flow inflates genetic variance and reduces local population density sufficiently that genetic drift overcomes local selection, creating a finite range margin. When a flat centre is superimposed on a linear gradient, gene flow increases genetic variation dramatically at its edges, leading to an abrupt reduction in density that prevents niche expansion. Remarkably local interruptions in a linear ecological gradient (of a width much less than the mean dispersal distance) can prevent local adaptation beyond this flat centre. In contrast to other situations, this effect is stronger and more consistent where carrying capacity is high. Practically speaking, this means that habitat improvement at patch margins will make evolutionary rescue more likely. By contrast, even small improvements in habitat at patch centres may confine populations to limited areas of ecological space.

## 1 INTRODUCTION

Why is local adaptation prevented in some ecological and genetic situations, meaning that populations cannot track changing environments, and so have finite ranges in space and time? Understanding when and where such limits to adaptation occur is critical for predicting species’ extinction rates in time, their geographical distributions in space, and the evolution of ecological communities. Information on maximum rates of evolution allows estimates of where and when rapid environmental change will cause the loss of species from ecological communities. Understanding how genetic and ecological processes interact also allows scientists to provide guidance on how to maximize evolutionary rates in populations that are close to critical rates of environmental change.

Single population models for the maximum sustainable rate of evolution (“evolutionary rescue” models; Bell, 2013) exclude the genetic and demographic effects of dispersal between ecologically divergent populations. The movement of individuals and alleles between different environments has two contrasting effects (Bridle, Polechová, & Vines, 2009; Bridle & Vines, 2007; Connallon & Sgro, 2018; Haldane, 1956): (a) it reduces population mean fitness, because the phenotypes of incoming individuals and their offspring are distant from the local optima (either by changing the trait mean or by increasing its variance, or both); and (b) it increases evolutionary potential by increasing local genetic variation.

Models exploring the effect of gene flow on local adaptation have focussed on either a few ecologically divergent patches (often with different carrying capacities), with varying levels of dispersal between them (see, e.g., Legrande et al., 2017 for a review); a series of populations with stepping‐stone dispersal (e.g., Alleaume‐Benharira, Pen, & Ronce, 2005); or the joint effect of gene flow and selection when individuals are distributed continuously across a linear ecological gradient in space (Barton, 2001; Bridle, Polechová, Kawata, & Butlin, 2010; Haldane, 1948; Kirkpatrick & Barton, 1997; Polechová, 2018; Polechová & Barton, 2015). These models of ecological margins explicitly couple population genetics with population ecology, in that the match of a genetically variable trait to the optimum determines individual fitness (see reviews by Bridle, Polechová, et al., 2009; Bridle & Vines, 2007; Kawecki, 2008; Lenormand, 2002). Dispersal along ecological gradients generates a fitness cost (termed “standing load”). When the trait mean matches the optimum, this load is the reduction in mean fitness that arises due to the increased phenotypic variation in the population that is generated by dispersal. This standing load reduces the rate of population growth. If the population mean also fails to match the local optimum, there is an additional “maladaptation load,” which increases with the mismatch between the trait mean and its optimum, and as the strength of selection increases. However, where all populations match the local trait optima, gene flow has no effect on the mean phenotype (although it still affects the variance) because alleles arrive and leave all populations equally, so that gene flow has no net effect on local allele frequencies (Felsenstein, 1975; Kirkpatrick & Barton, 1997). However, where there is a mismatch between the local trait mean and the local optimum (i.e., maladaptation load), asymmetrical migration is generated due to a gradient in density, with density being highest where the mean matches the local optimum. This increases maladaptation in populations at lower density and may cause local populations to collapse through a positive feedback between maladaptation, population density and asymmetrical gene flow. A finite range limit therefore forms. However, such a finite limit depends on populations being able to match the local trait optima in some parts of the range, but failing to do so in other parts of the range.

When genetic variance is not allowed to evolve as a result of dispersal along a linear gradient Kirkpatrick & Barton, 1997), three regimes emerge: “Unlimited Adaptation” (where the trait evolves to match the spatially changing selective optimum everywhere); “Limited Adaptation” (where the population is well adapted to the local optimum only at the centre of the species’ range); and “Extinction” (where the population cannot be sustained at any point on the gradient). “Limited Adaptation” behaviour is characterized by asymmetrical dispersal from the well‐adapted central region, where population density is high, to the poorly adapted margins. In “Unlimited Adaptation” behaviour, no density gradient is generated because the population is well adapted everywhere. Dispersal is therefore symmetrical across the range, allowing the population to expand in niche space (i.e., along the ecological gradient) without limit.

By contrast, allowing additive genetic variance to evolve due to dispersal between environments allows adaptation along virtually any steepness of ecological gradient, over a very wide range of conditions, and for a range of quantitative genetic models (Barton, 2001). Eventually, however, a deterministic limit is reached when the variance generated by dispersal reduces population mean fitness (i.e., growth rate) sufficiently to cause extinction throughout the whole range, despite allowing evolution of the trait mean to match the local optimum everywhere. At this deterministic limit, although the population has sufficient genetic variance to track the rapidly changing trait optima, the standing load caused by this amount of genetic variance reduces population growth to zero (so the population goes extinct everywhere across the range).

### 1.1 Effects of colonization and finite population size on maladaptation

Barton (2001) quantified the ecological and genetic conditions for which a population that begins perfectly adapted to a linear gradient can be sustained. However, his analyses did not include stochastic effects on either allele frequencies or population dynamics. Bridle et al. (2010) used individual‐based simulations to explore how the limits to local adaptation changed when a finite population colonized a linear gradient at its centre. In addition, they varied the maximum productivity (the “carrying capacity”) of all patches across the range to test the effect of population density on local adaptation. These simulations showed that: (a) local adaptation (and niche expansion) was prevented at a lower gradient steepness than predicted by deterministic models; (2) for most of parameter space, only two outcomes were observed along a linear gradient: extinction everywhere, or adaptation without limit. The failure to track a linear gradient was associated with reduced population density, caused by the evolution of genetic variance as gradient steepness increased. This suggested that the stochastic effects of finite population size prevent adaptation at ecological margins.

Polechová and Barton (2015) provided an analytical solution for the issue highlighted by Bridle et al. (2010). In particular, they demonstrated that local adaptation is prevented where population density is reduced below a critical point by the load imposed by the genetic variance generated by gene flow. Prevention of adaptation was therefore due to genetic drift overcoming selection rather than because of stochastic population dynamics. This critical limit is found without genetic constraints or fitness trade‐offs, where allele effects are unequal, and in the presence of epistasis. These conclusions have recently been extended to two‐dimensional environments (Polechová, 2018). Polechová and Barton's (2015) model therefore provides a general explanation for the failure for populations to adapt at a narrower range of parameter conditions (in terms of gradient steepness and population demography) than predicted by the deterministic limit, as observed by Bridle et al. (2010).

### 1.2 Limits to adaptation along nonlinear ecological gradients

At their most realistic, linear models of adaptation along ecological gradients typically produce only two outputs in most regions of parameter space: unlimited species’ ranges (adaptation everyone), or extinction (adaptation nowhere). This is in marked contrast to the limited ranges that are ubiquitous in nature. However, ecological gradients in nature are rarely linear, as perceived by the organisms themselves (and their alleles). Instead, they consist of patches of good habitat surrounded by habitat of rapidly decreasing suitability. For these reasons, ecologists have questioned the relevance of migration load, and the modelling of linear gradients, to global species’ margins in nature (e.g., Blows & Hoffmann, 2005; Holt, 2003; Holt & Keitt, 2005; Thomas & Kunin, 1999).

The study of nonlinear (“steepening”) gradients represents an important link between gradient models, where gene flow and its effects on genetic variance are an emergent property of population demography along an ecological gradient (e.g., Barton, 2001; Bridle et al., 2010; Polechová, Marion, & Barton, 2009), and patch models, where discrete patches differ in carrying capacity (and therefore density), and are subject to fixed probabilities of connection by dispersal (Legrande et al., 2017). Instead, real ecological margins are likely to involve both changes in the density of suitable patches, and variation in conditions within patches.

A patch can be modelled as an area in which ecological conditions change progressively with distance from the centre, with the rate of change increasing to the point where the patch margin is determined by the population's maximum rate of adaptation (see Butlin, Bridle, & Kawata, 2003). Nonlinear gradients will be close to reality in many situations, for example where multiple ecological factors interact at particular parts of an ecological gradient, or where the trait mean must change in a nonlinear way to match a linear gradient in some abiotic factor such as temperature, due to threshold or interacting effects with other factors, or the presence of other species (e.g., Case, Holt, McPeek, & Keitt, 2005). Modelling local adaptation along steepening ecological gradients also means that a stable margin is always predicted at a critical level of steepness, based on the deterministic predictions (Barton, 2001), as well as analytic predictions based on the effect of genetic load on the power of selection compared to genetic drift (Polechová & Barton, 2015).

In this paper, we explore the “critical drift” threshold for adaptation limits (Polechová & Barton, 2015) by extending the simulation model of Bridle et al. (2010) to test the effect of colonization and of different types of nonlinear ecological gradients on local adaptation. Firstly, we use a perfectly adapted starting population to eliminate stochastic effects arising from colonization dynamics and the establishment of phenotypic clines. This allows us to compare the demographic and ecological parameters required for a colonizing population to adapt along an ecological gradient with those that allow population persistence.

We then explore, for both these “colonizing” and “established” conditions, the effect on local adaptation of departures from linear ecological gradients using either: (a) “steepening” gradients, characterized by an ecological gradient that becomes increasingly steeper with distance from the centre and; (b) linear gradients with parameter conditions that generated unlimited spread in Bridle et al. (2010), but where the gradients are now interrupted by a flat central portion of variable width where the optimum phenotype does not change.

We show that the introduction of even narrow regions without change along a linear gradient prevents extinction. However, this flat region also generates small areas of high population density that create the asymmetries in gene flow that prevent adaptation at the patch edge, especially where maximum population sizes are large. This suggests that surprisingly local regions of shallow gradient within linear ecological gradients can generate narrow species ranges, even for parameter values that would allow adaptation along uniform linear gradients. This observation has implications for managing populations to maximize their evolutionary resilience.

## 2 THE SIMULATION MODEL

The basic model is identical to the individual‐based simulation described in Bridle et al. (2010). The evolutionary dynamics for the simulated population take place within a continuous region of maximum extent 32,000 × 1,000 units. There is an ecological gradient along the long (*x*) axis, which is uniform with slope *b*. The area is simulated as a cylinder; the edges of the second, short (*y*) axis are joined. Individuals occupy the vertices of a grid and more than one individual can occupy any given position. The model either (a) follows the fate of a starting population of 500 individuals that are initially distributed in the central 500 × 1,000 units of the environment (“colonizing start”); or (b) introduces a population that is fully adapted across the entire gradient, and allows the simulation to run from that point to test its stability (“perfect start”).

The phenotype is determined by diploid unlinked bi‐allelic loci with additive effects that mutate symmetrically at rate μ (μ = 0.0001 per locus per generation unless otherwise stated). For all runs, 64 loci were used, with allelic effect α = 1 (maximum phenotypic range = 0–128). Population growth is logistic, dependent on the local density of individuals (*N*) and local carrying capacity (*K*).

For the colonizing start (initially *N* = 7.85 individuals), individual phenotypes range from *z*_opt_ − 2α to *z*_opt_ + 2α where *z*_opt_ is the optimum phenotype at the centre of the range For the perfect adaptation start, the population density was set at carrying capacity throughout the range and spatial positions were drawn randomly from a uniform distribution. Genotypes for these perfectly adapted individuals were generated on the basis of cline widths and spacing predicted by Barton (2001) using C++ and R scripts available on request from the authors.

Females choose mates from the males available within a finite mating distance (MD), with a probability proportional to the fitness of each male at its position on the ecological gradient. This was fixed at MD = 150 (see Butlin et al., 2003 for a description of the effect of male dispersal on range expansion). Offspring then disperse and viability selection occurs after dispersal through the number of offspring produced by each female. If no male is available within the mating area, the female leaves no offspring.

The offspring of each female disperse to new positions in the habitat with a Gaussian distribution of dispersal distances, mean 0 and standard deviation *D*, in uniformly distributed random directions. Since mating is a form of dispersal by males (or their gametes), the standard deviation of total dispersal is given by $\text{TD}=\sqrt{D^{2}+\frac{1}{2}{\text{SM}}^{2}}$ (see Crawford, 1984), where SM is the expected distance between mating partners when a female chooses from a circle with radius MD, hence SM = (MD/√2). The expected distance σ along the *x*‐axis is only in one dimension, hence σ = TD/√2.

The fitness of both sexes is determined by the same function. The number of offspring that a female leaves is drawn from a Poisson distribution with mean *W*_F_ = 2 + *r*_F_ (1 − *N*/*K*) − s (*b* *x* − *z*)^2^/2 (*W* ≥ 0). In our model, there are no random effects on death rates, or selective mortality. These are determined precisely by the ecological gradient and the local population density relative to *K*. The maximum rate of increase *r*_m_ = *r*_F_/2; *r*_F_ is set to 1.6. *K* is the carrying capacity within a circle of radius 50 around the focal individual, *N* (density) is the number of individuals in such a circle. *U_x_* = *b* *x* is the phenotypic optimum at the point (*x*) on the gradient occupied by the female. The parameter *s* measures the rate of decline in fitness for phenotypes that depart from the optimum; the strength of stabilizing selection *V*_S_ is 1/(2s). Here, *V*_S_ is set to 4 and b (the spatial gradient in the optimum) is either set to 0.004, determined by the sigmoid function, or interrupted by a flat central region.

Note that when drift and the effects of the margins are negligible, increasing dispersal with constant gradient is equivalent to increasing the gradient with constant dispersal: by dispersal of a distance σ, fitness decreases by

$$\frac{b^{2}\sigma^{2}}{2V_{\text{S}}}$$

The growth rate of a particular phenotype is

$$r\left[z,N\right]=r_{\text{m}}\left(1-\frac{N}{K}\right)-\frac{{\left(z-U_{x}\right)}^{2}}{2V_{\text{S}}},$$

and hence, the growth rate of the population is the average over all phenotypes

$$r_{N}=〈r[z,N]〉=r_{\text{m}}\left(1-\frac{N}{K}\right)-\frac{{\left(\overset{¯}{z}-U_{x}\right)}^{2}}{2V_{\text{S}}}-\frac{V_{\text{P}}}{2V_{\text{S}}}$$

assuming additive genetic variation, and no environmental or genetic variation in phenotype.

For an infinite population, the population dynamics should approximately match the continuous time model described by equation 7 in Kirkpatrick and Barton (1997), and if no linkage disequilibria (LD) are generated, the evolution of phenotype should follow the two‐allele *n*‐loci model of Barton (2001). In our model, population regulation occurs over discrete generations, and populations are finite in size, therefore allowing stochastic effects on demography and allele frequency, and the generation of lags.

**Figure 1:**
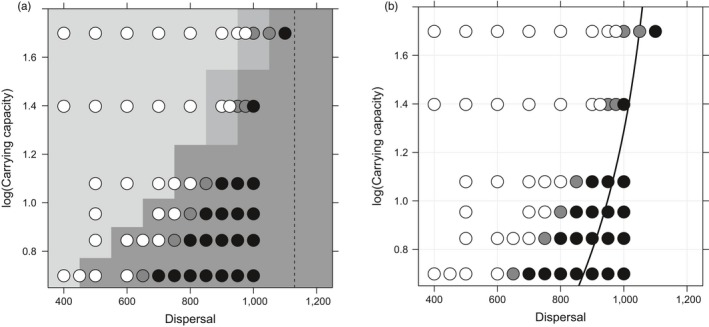
Evolution along a linear ecological gradient. (a) Compares outcomes from different starting conditions. The grey‐scale background summarizes data from the “colonizing start” runs of Bridle et al. (2010), showing outcomes at generation 3,000 for five runs for each parameter combination, for populations initially occupying only the centre of the gradient: light grey—unlimited spread, mid‐grey—limited spread, dark‐grey—extinction. The points represent outcomes for 3–5 runs, starting with a population occupying the whole area and perfectly adapted to the local optimum at each point: white—environment fully occupied, black—extinct, grey—mixed outcomes, including cases where the population fragmented. The dashed line is the deterministic limit of spread (where population density is reduced to zero by variance load, even when the phenotypic mean matches the optimum; Barton, 2001). (b) Compares “perfect adaptation” runs with the threshold prediction from Polechová and Barton's (2015) model (solid line), where $B=0.15N\sigma \sqrt{s}$ (*B* is the effective environmental gradient, *N *is the local population size, σ is the dispersal distance, and *s* is the selection per locus). The population is expected to persist only for dispersal distances below this threshold. In these simulations, the focal individual was removed from the calculation of local density for comparison with the Polechová and Barton (2015) model. Points filled as in (a). *K* is on log_10_ scale in both figures

**Figure 2:**
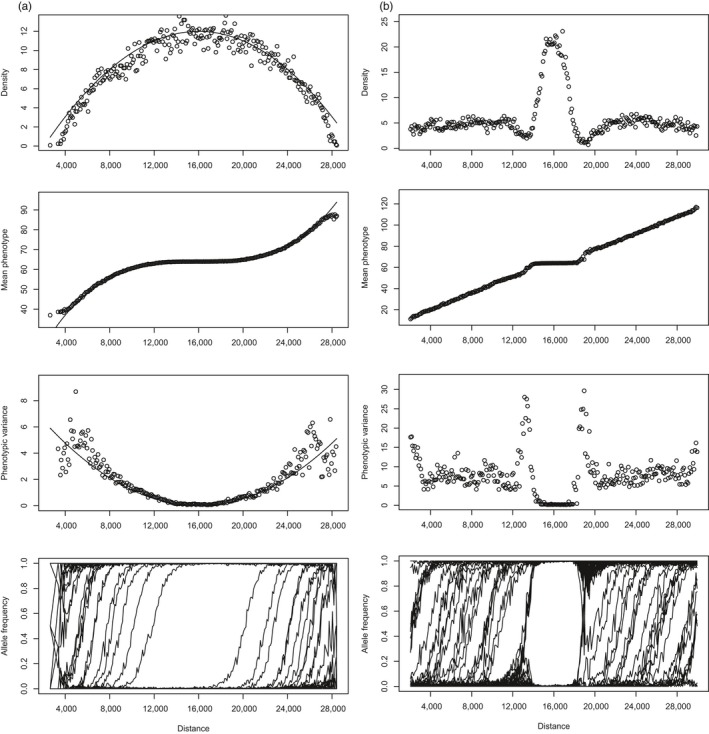
Example plots of “colonizing start” simulations at generation 3,000 for (a) a steepening and (b) a flat‐linear gradient, with spatial plots of phenotypic mean and variance, mean density and allele frequencies estimated from spatial slices of 100 units for carrying capacity *K* = 25 and dispersal TD = 500 for (a); K25 and dispersal TD = 850 with a flat centre width of 2,000 for (b). Predicted values for (a) from Barton (2001) are shown as solid lines

**Figure 3:**
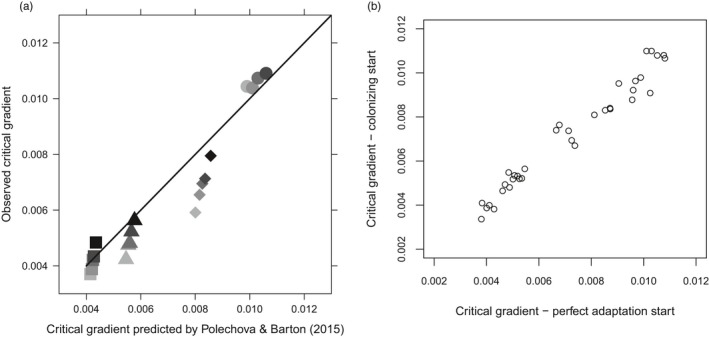
(a) Critical gradients that define population margins on a steeping gradient. The critical gradient predicted by Polechová and Barton (2015), as in Figure 1b, is compared to the critical gradient observed in our simulations for different carrying capacities, *K*, and dispersal, TD. Darker points indicate increasing *K* (5; 7; 12; 25); circles—TD = 400; diamonds—TD = 500; triangles—TD = 750; squares—TD = 1,000. (b) Comparison of critical gradients for parameter combinations in (a) after 3,000 generations when simulations are run from a “perfect adaptation” start compared to from a “colonizing start”

The program was written in C++, developed from that introduced by Kawata (2002) and is available on request from the authors. Output from the simulations for a given generation was analysed using a script in R, which calculated genetic variance, cline widths for each locus, population density and the distribution of phenotypes for a given portion of the range. The scripts are also available on request from the authors. To allow direct comparison to the predictions of Polechová and Barton (2015), the focal individual was removed from all calculations of population density for the runs shown in Figures 1b, 2a and 3, but it was retained elsewhere for comparison to Bridle et al. (2010). Note that (regardless of the form of the gradient), the carrying capacity (*K*) remains constant throughout the potential geographical range, so that gene flow will remain symmetrical across all species’ ranges, provided the optimum is matched everywhere.

### 2.1 Nonlinear ecological gradients

We extended our linear models to explore adaptation along two types of nonlinear gradient:

1. *Steepening gradient: *a sigmoid rather than linear gradient in selective optimum, where, the optimum phenotypic value changes with the cube of distance from the centre of the simulated range. Here, the uniform gradient in the phenotypic optimum (U_x_ = 0.004x) was replaced with a power relationship:

$$U_{x}=64+\frac{1.56{\left(x-16000\right)}^{3}}{10^{11}}.$$

This differs from the relationship used by Polechová and Barton (2015) and Polechová (2018) which was exponential in form with nonzero central slope. However, since both represent smoothly increasing rates of environmental change, we do not expect this difference to influence the threshold gradient at which further adaptation is prevented.

- *Flat‐linear gradient: *a uniform gradient of steepness *b* = 0.004 (i.e., identical to the steepness of linear gradient used by Bridle et al., 2010), which is interrupted by a central flat portion (*b* = 0) of width (*w*).

For (1), we explored the effect of various parameter combinations on the critical gradient, defined as the point at which the intrinsic rate of increase is zero, given the local distribution of phenotypes (*r*_F_ = 2 for *N* = 0, measured at generation 3,000). For (2), we explored the parameter combinations of carrying capacity per cell, *K* = 5–50, dispersal, TD = 400–1,100, and the width of the central flat portion, *w* = 0–4,000, that allow successful colonization of the patch centre, and then subsequent spread throughout the range. The majority of simulations were run for 3,000 generations, although the behaviour of some parameter combinations was tested for up to 10,000 generations.

## 3 RESULTS

### 3.1 Colonization and local adaptation along linear ecological gradients

Figure 1a shows that range collapse occurs even from a perfectly adapted start, for a similar range of parameter combinations to that observed by Bridle et al. (2010) when populations were established from a central starting position. Populations on a linear gradient of *b* = 0.004, begun from a perfectly adapted condition, can persist dispersal (TD) less than 1,100, but collapse quickly with TD more than 1,200, regardless of the value of carrying capacity, *K*, or the size of the starting population (Figure 1a). At TD less than 1,100, persistence depends on *K*. Extinction still occurs from a perfect start, as observed from colonization, suggesting that for much of parameter space, stochastic effects during colonization do not affect niche expansion. However, perfectly adapted populations within the area of parameter space that led to “limited adaptation” (Bridle et al., 2010) fragment and collapse as the trait cline becomes increasingly shallow relative to the gradient in the optimum. The process of range collapse from a perfectly adapted start in this region of parameter space can take more than 10,000 generations.

The simulated behaviour of populations along linear gradients away from the deterministic limit is qualitatively similar to the critical gradient predictions of Polechová and Barton (2015) (Figure 1b). However, extinction occurs in our simulations at lower values of dispersal and carrying capacity, associated with the higher variance and lower density we observe at these parameter combinations (Bridle et al., 2010). Polechová and Barton's (2015) analytical predictions suggest that limited adaptation should be stable for a small region of parameter space. However, we do not detect such a region of parameter space in our simulations.

### 3.2 Colonization and adaptation along steepening gradients

For colonizing start conditions, models of adaptation along steepening ecological gradients explore the establishment and growth of a population to occupy a patch of suitable habitat. As the population expands through habitat at the centre of the patch, where the environment changes gradually in space, its continued growth depends increasingly on its ability to adapt at the margins, where the environment changes rapidly.

For both steepening and flat‐linear forms of nonlinear gradient (Figure 2a,b, respectively), the starting population is well adapted to the central part of the range, and so should quickly colonize and expand throughout the shallow or flat, central portion. However, for a steepening gradient expansion should always stop at some distance from the centre, where a critical rate of ecological change is reached. By contrast, in the case of a flat‐linear gradient the population should continue to expand along the linear part of the gradient provided it can evolve to pass the sudden change in gradient at the edge of the flat portion. Note that for all of our flat‐linear runs, we use values for dispersal and carrying capacity that allow unlimited adaptation along a completely linear gradient of the same steepness. This allows us to assess the effect of an abrupt change in the gradient on local adaptation.

#### 3.2.1 Colonization and adaptation along steepening gradients

As seen along linear gradients (Bridle et al., 2010; Figure 1), the critical gradient at which adaptation fails increases as total dispersal decreases and as population density (determined by the carrying capacity, *K*) becomes greater (Figure 3a). The value of this critical gradient does not differ when populations colonise the centre of the patch and spread as they adapt, compared to when they are perfectly adapted along the entire ecological gradient at the start (Figure 3b).

Figure 3a compares our simulations to predictions for the critical limit in Polechová and Barton's (2015) model. The simulated values again show a qualitative pattern that follows analytical predictions, although the quantitative mismatch tends to increase with lower carrying capacity (*K*).

#### 3.2.2 Colonization and adaptation along a flat‐linear gradient

**Figure 4:**
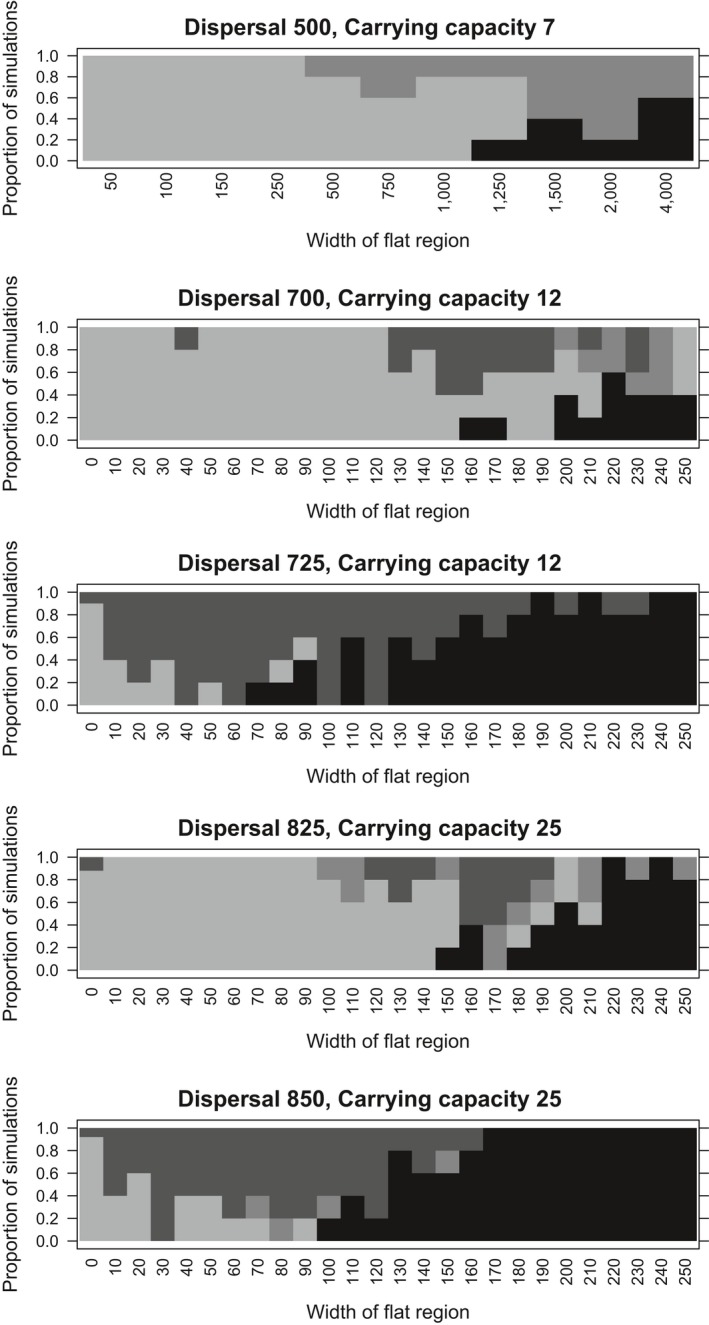
The effect of a flat portion in the ecological gradient of width (*w*) on range expansion (from colonization) at generation 3,000. The behaviour of the simulations is summarized as follows: (i) LIGHT GREY: full spread; (ii) DARK GREY: slow spread (a phenotypic cline forms but has not spread outside 7,000 < *x *<25,000 by generation 3,000); (iii) MEDIUM GREY: spread on one side only (phenotypic cline forms but only on one side of the gradient); (iv) BLACK: no spread (population remains confined around flat portion of gradient, with no phenotypic cline)

Populations along flat‐linear gradients differ from a steepening gradient in that the shift in gradient steepness is abrupt (it is an interruption of an otherwise linear gradient). These simulations show that a remarkably small flat central portion (*w*) consistently prevents adaptation from colonization for up to 10,000 generations for wide regions of parameter space (Figure 4), particularly where carrying capacity, *K*, is high. For example, with dispersal TD = 850, carrying capacity *K* = 25 (Figure 4; bottom panel), a flat centre of only *w* = 100 units (1/8 of the mean dispersal distance) can prevent spread of the population from the centre, generating a finite range margin. Along interrupted gradients, therefore, higher population density prevents local adaptation at the edges, rather than making it more likely, as is the case for the steepening gradient.

The constraining effect of interrupting the ecological gradient is reduced at lower values of carrying capacity and dispersal. For example, at *K* = 7, TD = 500 (Figure 4; top panel) the width of the flat portion needs to be about *w* = 2,000 units to prevent spread from the range centre (i.e., four times the mean dispersal distance). By contrast, at *K* = 12, adaptation from the flat centre is consistently prevented even when its width is ¼ of the dispersal distance (e.g., at TD850 and K25, a central width of 200 typically prevents local adaptation). At lower carrying capacity and dispersal values, greater variance in outcome is also observed among simulations for the same parameter values (Figure 4). For example, the population might spread to only one side of the gradient, on both sides, or on neither side in the time available for each simulation (typically 3,000 generations).

Note that although a remarkably small interruption to the linear gradient can prevent local adaptation from the centre, it also prevents extinction. Even when dispersal is high, the central portion remains occupied when additional simulations were conducted at dispersal distances that would cause rapid extinction everywhere in linear models (Figure 1a). Central population density can also rise to about twice that seen for the same dispersal and carrying capacity on the linear gradient, because there is no increase in genetic variation (and standing load) associated with dispersal at the flat centre.

### 3.3 Comparing colonization versus persistence along a flat‐linear gradient

Populations remain fully adapted for at least 3,000 generations if they are started from a perfectly adapted population, even for parameter combinations that fail to spread from colonization. Where these perfectly adapted runs are allowed to continue for up to 10,000 generations, populations sometimes fragment at the edges of the flat section for some parameter combinations (especially where *K* and TD are high), although gene flow between populations typically prevents this fragmentation lasting for long. This suggests that although the flat central portion has systematic effects on local adaptation from colonization, it only rarely causes range collapse when populations are initially fully adapted to the entire gradient.

**Figure 5:**
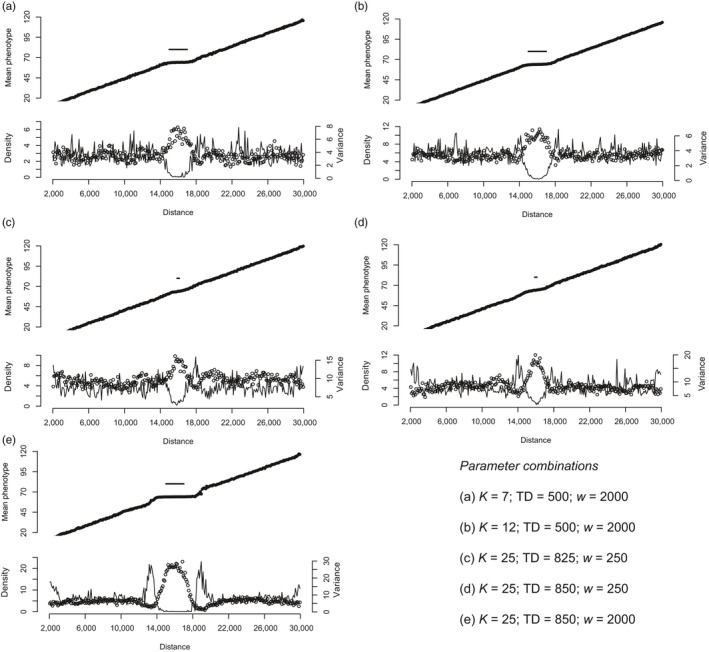
Patterns of phenotypic mean, density and genetic variance in simulations from perfectly adapted start on ecological gradients with a flat central portion, illustrating the increase in variance near the edge of the flat area and its effects on adaptation and population density for different combinations of width (*w*), carrying capacity (*K*) and dispersal (TD). Upper panels: The position and width of the central (flat) portion (*w*) is shown by a solid line above the observed trait mean. Lower panels: Local density is shown as open circles, local genetic variance by a solid line. Note that, apart from (b), none of these parameter combinations were able to fully spread from a colonising start (see Figure 4)

Simulations of flat‐linear gradients from perfectly adapted start allow exploration of the reasons why populations fail adapt from colonization even with low widths of the flat central region relative to total dispersal. In parameter regions where colonization and subsequent local adaptation is prevented by the flat centre, there is an inflation of variance at the point where the gradient steepens, due to the stronger effect of gene flow at this position. This reduces population density, generating a density trough at these points (Figure 5). This effect is greater for higher dispersal (compare Figure 5c and d), and less clear at lower values of carrying capacity (compare Figure 5a and b). It is difficult to observe at values of *w* where spread is only sometimes prevented (e.g., for TD = 500, *K* = 7, *w* = 100: Figure 5a compared with Figure 4 top panel). However, the density trough generated by the stepped gradient becomes very marked when the flat centre is wider and carrying capacity is high. For example, in Figure 5e, where *w* is more than twice the mean dispersal distance, gene flow not only inflates variance and so reduces local population density (so increasing drift), it also generates maladaptation load due to the mismatch of the local trait mean to the optimum. In addition, as the flat centre widens (*w* increases), the population density at the range centre approaches the carrying capacity (*K*), even when dispersal is high. The dynamics of these models therefore differ from the linear gradient, where dispersal has a similar effect on variance (and therefore population density) at all points along the range.

## 4 DISCUSSION

We have extended our individual‐based simulations (Bridle et al., 2010) to include tests of the stability of populations that are already perfectly adapted everywhere on an ecological gradient, across a range of gradient steepness, population density, and mean dispersal. This approach tests the role of stochastic ecological and evolutionary processes on the failure of populations to adapt along ecological gradients. We have also determined the parameter conditions that cause maladaptation along two forms of nonlinear gradient, and compared results for one of these to the analytical predictions of Polechová and Barton (2015). Below, we discuss these results and consider their implications for practical interventions to increase evolutionary potential in populations and therefore the resilience of ecological communities to environmental change.

### 4.1 Adaptation along linear gradients

Simulations that begin from a perfectly adapted state rapidly (typically within 500 generations) collapse in almost all the parameter combinations that showed “extinction” behaviour from a colonizing start (Figure 1a). Similarly, perfectly adapted populations were stable in parameter space that previously generated “full adaptation” behaviour (Bridle et al., 2010). Perfectly adapted populations took longer to fragment and collapse close to boundary conditions, and only collapsed after 10,000 generations for the large carrying capacity, high dispersal parameter combinations that characterized the (small) region of “limited adaptation” parameter space from colonization. In this region, populations always (eventually) collapsed throughout their range, rather than forming the long‐lived but finite species’ ranges observed in Bridle et al. (2010). This result contrasts with Polechová and Barton (2015), who observe a small region of parameter space that generates stable, finite ranges. Our result suggests that in our colonizing simulations, finite ranges (“limited adaptation”) are a product of stochastic processes, for example, in the establishment of clines in allele frequency during range expansion.

Overall however, the close match of these “perfect start” simulations with our “colonizing start” simulations indicates that the large area of parameter space where extinction is observed (Figure 1) cannot be explained by stochastic processes associated with range expansion. Instead, failure to establish or maintain local adaptation (and broader niches) is due to the inability of selection to overcome genetic drift where population density is reduced beyond a critical point by migration load (Bridle et al., 2010; Polechová & Barton, 2015). Even with stochastic effects on allele frequency and demography, linear ecological gradients cannot easily explain limited ranges in nature.

Our linear gradient simulations qualitatively match the “critical gradient” predictions of Polechová and Barton (2015), although maladaptation occurs at slightly lower gradient steepness and population density (Figure 1b). This may be due to nonuniform distributions of individuals, reduced selection on males, and/or failure of females to mate at low densities, given that genetic variance was higher and density lower in our simulations compared to analytical models (Bridle et al., 2010). We previously speculated that this inflated genetic variance was due to greater than expected linkage disequilibrium, based on values for LD that we estimated before rather than after selection had occurred. However, according to Felsenstein (1976), linkage disequilibrium should return almost to zero following selection, meaning that this effect cannot explain our higher than expected levels of genetic variation.

Polechová and Barton (2015) explore limits to local adaptation in one‐dimensional, rather than two‐dimensional space, where the effects of drift and dispersal, and their interaction with selection will differ (see Barton, Depaulis, & Etheridge, 2002). Polechová (2018) extended her critical gradient predictions to two‐dimensional habitats, showing that dispersal in the second dimension weakens the effect of drift, so allowing adaptation at a greater range of parameter values (i.e., moving the critical carrying capacity to the right of the predictions in Figure 1b). This effect of increased dimensionality is therefore in the wrong direction to explain the discrepancy between our simulations and Polechová and Barton (2015), meaning that the mismatch must be due to other differences between the models. However, qualitative agreement between our models remains in that increasing population size and reducing gene flow makes local adaptation overall more likely.

### 4.2 Adaptation along steepening gradients

A similar result to the linear gradient was obtained where the ecological gradient was given a sigmoid (steepening) shape (Figure 2a). A critical limit for niche expansion is always reached at some point beyond the less‐rapidly changing centre of the range. As above, for steepening gradients, the behaviour of our simulations from perfect start (Figure 3b) is equivalent to that observed from colonization (Bridle et al., 2010), even when simulations are run for up to 10,000 generations. Again, this result indicates that the failure to adapt (or to maintain an adapted state) at ecological margins results from a failure of selection to overcome drift caused by migration load, rather than from the stochastic effects of colonization, cline establishment or population dynamics. In contrast to the linear gradients, however, a finite species range is always observed (rather than extinction everywhere), because populations can always persist close to the range centre, where the ecological gradient is relatively shallow. In these regions, populations maintain sizes that are close to the carrying capacity of each patch, because the inflation of genetic variance due to gene flow within these shallow regions of ecological change is low, so reducing migration load (Figure 2a).

Figure 3a shows the match of our steepening gradient simulation outcomes to the Polechová and Barton (2015) one‐dimensional critical gradient prediction. As with linear gradients, our simulations show a qualitative match, in that higher population densities and reduced dispersal allow the population trait mean to track steeper local ecological gradients. This means that local adaptation is prevented closer to the patch centre when the habitat carrying capacity (*K*) (and therefore the population density) is lower, and dispersal (TD) is higher. However, along steepening gradients, maladaptation occurs at relatively lower values of dispersal and carrying capacity than along linear gradients, because of increased density at the centre causing asymmetrical gene flow (and therefore swamping of local adaptation), at the point where the population fails to track the local optimum. The quantitative mismatch between our simulations and analytical models (Polechová & Barton, 2015) is generally in the same direction as for linear gradients, and so is also unlikely to be explained by dimensionality. However, increasing carrying capacity tends to reduce this mismatch (Figure 3a). This suggests that stochastic effects in regions of low population density, possibly due to nonuniform distributions of males and females, increase extinction risk in our simulations.

### 4.3 Adaptation along flat‐linear (interrupted) gradients

In contrast to the gradients explored above, stochastic population processes during expansion consistently prevent local adaptation along linear gradients that are “interrupted” by a region of habitat where the required trait mean does not change. Within these regions, gene flow does not inflate genetic variance (or create standing load), generating densities around the patch centre that almost match the local carrying capacity (Figure 2b). In turn, this generates strong asymmetrical gene flow outward from the centre that imposes a load on edge populations. In natural populations, such regions of reduced environmental change might be generated by a mountain ridge, or area of deep water, superimposed on a latitudinal gradient, or an area of habitat where a predator or competitor has been excluded (Svenning et al., 2014).

Spread from colonization can be prevented by a remarkably small interruption to an ecological gradient, even for as long as 10,000 generations (Figure 4). However, when run from a perfectly adapted start, these populations remain stable for up to 10,000 generations. This represents an important contrast with the other gradient types, suggesting that stochastic effects prevent expansion from the range centre. Another important contrast is that extinction never occurs at the range centre (i.e., on the flat central portion) for the parameter combinations tested. Flat‐linear gradients therefore provide stable patches for population persistence, while preventing local adaptation at their edges for a wider range of conditions than linear gradients. These simulations therefore predict (somewhat counter‐intuitively) that improved conditions at the centre of a patch, or a region where the ecological gradient becomes shallower (e.g., a region of reduced dispersal, or an elevational gradient embedded within a latitudinal gradient) may prevent niche expansion through local adaptation at the patch edge.

Surprisingly, increasing carrying capacity has a qualitatively different effect along a flat‐linear gradient than along a steepening or linear gradient. Instead of facilitating niche expansion, adaptation along a flat‐linear ecological gradient is apparently more difficult at higher carrying capacities. For example, at *K* = 7, with dispersal TD = 500, the width of the flat portion needs to be at least 1,250 units to prevent spread from the range centre (i.e., 2.5 times the mean dispersal distance) (Figure 4, top panel). By contrast, at carrying capacity *K* = 25 and dispersal TD = 850, a flat centre of width only 100 units (i.e., c. 1/8 mean dispersal) prevents expansion across the entire range from a colonizing start (Figure 4, bottom panel). This is despite the fact that perfectly adapted runs are stable at both these parameter conditions. This contrasting effect of stochastic processes may be because a greater and more consistent difference in density from the centre to the edge is achieved at higher carrying capacity and dispersal (Figure 5a vs. e), leading to more consistently asymmetrical gene flow that more effectively swamps local adaptation at the edges. Along flat‐linear gradients, therefore, stochastic effects may favour local adaptation and niche expansion, by weakening the swamping effects caused by the local inflation of genetic variance by dispersal, and consequent increase in standing load. Abrupt changes along ecological gradients (i.e., at habitat patch edges), therefore seem highly effective in preventing adaptation. This is true even for patches that are small relative to total dispersal. For example, our simulations suggest that such patches need only be a quarter the width of mean dispersal per generation, provided the patches can support a high local population size at their centre. Limits to evolutionary responses caused by such nonlinear gradients may therefore be especially pervasive for small, relatively mobile organisms, such as butterflies.

The predictions for steepening gradients from Polechová and Barton (2015) cannot be applied directly to the flat‐linear gradients, because these estimate critical gradients based on a smooth increase in the rate of environmental change, not on the effects of abrupt changes in gradient.

### 4.4 Relevance of local adaptation in patches to global range dynamics

The types of gradient modelled here are more likely to reflect local patch dynamics than global range dynamics. Populations within patches may adapt to local conditions but be trapped by steepening gradients as they expand away from the centre. Patches may also differ in overall quality, reflected by their carrying capacity. This means that the evolutionary dynamics of local patches, each determined by the interaction between genetic load, genetic variation and population demography, will drive larger scale patterns of species’ persistence, particularly during responses to ecological change.

Steepening gradients provide an important bridge between population genetic models of adaptation along uniform gradients and ecological reality, where environmental gradients are complex in form, but are generally steeper at patch margins than within patches. At a range margin, there is also a larger scale gradient in patch availability, size and connectedness, but these parameters are not constants. Instead, they depend on local adaptation within patches. The more productive the patch, the greater the density in the centre and therefore the further out along the ecological gradient the patch margin will form. This larger (and more ecologically resilient) patch will therefore act as a more effective source of colonists for other patches because of the greater number of individuals and the wider range of genotypes it supports. This is because new patches can only be colonized successfully if a nearby patch has sufficient genetic variation to host phenotypes sufficiently close to the optimum to colonize the new patch.

### 4.5 Empirical tests of these models

An important prediction of our simulations and those of Polechová and Barton (2015), and Polechová (2018) is that we should observe relatively little maladaptation across a species’ geographical range. Instead, the genetic variation generated by gene flow should allow a population to track the local trait optima effectively (e.g., Fitzpatrick et al., & Reid, 2019) until some critical gradient is reached where the population collapses, generating either an abrupt edge in the case of a nonlinear gradient, or extinction everywhere along a linear gradient. Standing load (and genetic variance) should therefore increase (and local density decline) as the margin is approached, even though the trait mean matches the optimum. However, because the population density will be low where variance causes population collapse, empirical estimates of genetic variance, density and trait mean are challenging (Bridle, Gavaz, & Kennington, 2009; O'Brien, Higgie, Reynolds, Hoffmann, & Bridle, 2017). In our flat‐linear simulations, we can infer the critical values for these parameters only because populations that fail to spread from colonisation (Figure 4) persist when started from a perfectly‐adapted state (Figure 5). The environmental sensitivity of genotypes (plasticity) may also increase or reduce phenotypic variance in different parts of the gradient (Saxon, O'Brien, & Bridle, 2018), potentially increasing standing load, without necessarily increasing evolutionary potential (Chevin, Collins, & Lefevre, 2013).

Our simulations demonstrate that stochastic effects along nonlinear gradients have contrasting effects depending on how and where the gradient changes. Along steepening gradients, increasing carrying capacity increases the critical gradient at which adaptation fails (Figure 3a), and so allows the population to expand its niche further into the steepening regions of ecological change at the patch edge. By contrast, where a linear gradient is interrupted by a flat region where gene flow generates no standing load, stochasticity makes niche expansion more likely, apparently by reducing the strength and consistency of the density gradient (and swamping effect) generated by the abrupt change in the ecological gradient.

### 4.6 Maximizing evolutionary responses in natural populations

Our simulations, and similar models, make important simplifying assumptions. In particular, our ecological gradients remain constant over 100s or 1,000s of generations. Although this might be reasonable for some abiotic gradients, it is less likely where species’ interactions are strong (e.g., Case et al., 2005; Singer & Parmesan, 2019), where interactions occur between alleles from different species (e.g., Svensson & Connallon, 2019), or where behavioural responses of organisms may smooth or steepen ecological gradients locally (Nadeau, Urban, & Bridle, 2017a, 2017b). In particular, rapid changes in biotic interactions (e.g., due to prey‐switching or host‐switching by predators or pathogens in some regions of climatic space) could quickly create nonlinear ecological gradients. Nonlinear ecological gradients will also occur where changes in some values for a given trait require more allelic substitutions than others, because of nonadditive genetic effects (Butlin et al., 2003; Savolainen, Lascoux, & Merilä, 2013).

It remains difficult to obtain empirical measurements of the ecological and genetic parameters necessary to predict where range margins might occur. Nevertheless, our simulations do suggest some principles for managing populations to maximize their evolutionary potential. In particular, the surprising sensitivity of population density to asymmetrical effects generated by varying gradients in space, even on scales below the dispersal distance, suggests that improvements in patch centres might *reduce* adaptation towards patch margins, compromising the persistence of a population in a patch, and its potential to act as a source of colonists for other patches. Improving habitat at patch centres might therefore reduce the geographical range of target populations. Assisted migration and habitat improvements should therefore focus on patch margins rather than on their centres, which would reduce the density gradient (and asymmetry in gene flow) at the patch edge, so helping populations to adapt past the demographic sink created by an abrupt change in the environmental gradient.

Such efforts would be especially important for organisms where the ecological gradient varies in space at a fine scale relative to individual mobility, and where local population densities can vary widely. Similarly, for steepening (but continuous) gradients, if conservation efforts focus on improving conditions at patch centres, gradients at the edge will be made steeper which will reduce the occupied area. Therefore, for both flat‐linear and steepening gradients, improving the margin, even if it cannot be brought to the same quality as the centre, will increase the area of ecological space occupied by the population. This will, in turn, increase genetic variation at the centre (even if it reduces central density), so increasing evolutionary resilience to ongoing environmental change and the potential of the patch to act as a source for (re)colonization of other ecological patches.

## CONFLICT OF INTEREST

None declared.

## DATA ARCHIVING STATEMENT

Simulation and analysis code are available from the authors.
